# Elevating e-government: Unleashing the power of AI and IoT for enhanced public services

**DOI:** 10.1016/j.heliyon.2024.e40591

**Published:** 2024-11-20

**Authors:** Abdullah M. Al-Ansi, Askar Garad, Mohammed Jaboob, Ahmed Al-Ansi

**Affiliations:** a College of Commerce and Business Administration. Dhofar University, Salalah, Oman; b Faculty of Economic and Business, Universitas Muhammadiyah Yogyakarta, Yogyakarta, Indonesia; c Deputy Vice Chanceller Office, Dhofar University, Oman; d Faculty of Engineering, Thamar University, Yemen

**Keywords:** Artificial intelligence, Internet of Things, E-government, Digital government, Public administration, Public services, Citizen engagement, Service delivery

## Abstract

This study aims to explore how e-government services can be enhanced through the application of artificial intelligence (AI) and the Internet of Things (IoT). Specifically, it seeks to identify key themes, trends, challenges, and opportunities surrounding the integration of AI and IoT technologies in e-government, with a focus on understanding their implications for service delivery, governance practices, and citizen engagement. A systematic review approach was employed to analyze scholarly articles published between 2014 and 2024, sourced from various academic databases. The review encompassed studies exploring the adoption, implementation, and impact of AI and IoT in e-government contexts. Content analysis, thematic synthesis, and theoretical frameworks were utilized to distill insights and draw conclusions from the literature. The analysis revealed several important findings regarding the role of AI and IoT in enhancing e-government services. Key themes identified include the potential of AI and IoT to improve decision-making processes, optimize service delivery, and foster citizen engagement. However, challenges such as data privacy concerns, ethical considerations, and socioeconomic disparities in access were also identified. The study provides theoretical insights into the evolving landscape of digital governance and offers practical recommendations for policymakers and practitioners. This study contributes to the existing literature by offering a comprehensive analysis of the implications of AI and IoT adoption for e-government practices. It synthesizes findings from a diverse range of scholarly articles, providing insights into the complexities of digital transformation in the public sector. Theoretical frameworks such as ethical AI principles and digital governance models are employed to elucidate the implications of AI and IoT integration for public administration and governance. The findings of this study hold significant value for scholars, policymakers, and practitioners interested in understanding the impact of AI and IoT on e-government services. By highlighting key themes, trends, challenges, and opportunities, this study informs evidence-based decision-making and guides future research and policy development in the field of digital governance. Ultimately, it contributes to the advancement of knowledge and practice in leveraging AI and IoT technologies to enhance e-government services in the digital age.

## 1 Introduction

The incorporation of artificial intelligence (AI) and the Internet of Things (IoT) into e-government services has become a fundamental aspect of contemporary administration in the ever-changing digital world. AI-driven apps and Internet of Things (IoT) devices are leading this paradigm change, which is transforming how governments engage with citizens and allocate public resources. From AI-powered chatbots providing personalized assistance to IoT sensors enabling real-time data collection, these technologies promise to streamline operations, optimize decision-making, and improve service delivery [1]. However, as governments navigate this digital frontier, they must grapple with a host of complex issues, including data privacy concerns, ethical considerations, and socioeconomic disparities in access. E-government, a pivotal component of digital governance, encompasses a wide array of online services and platforms offered by governmental organizations to facilitate interactions with citizens, businesses, and other stakeholders [2].

The convergence of AI and IoT offers a revolutionary chance to improve these services' efficacy and delivery, spurring innovation and raising governance standards all around [3]. Artificial intelligence (AI) has the potential to improve decision-making in the public sector by streamlining administrative procedures, personalizing service delivery, and automating activities. It can evaluate enormous volumes of data [4]. Similarly, IoT technologies, by enabling connectivity and data exchange between devices and systems, empower governments to create smarter, more responsive infrastructures and deliver innovative services tailored to citizens' evolving needs [5].

Furthermore, the integration of AI and IoT in public administration has revolutionized how governments operate, making processes more efficient and improving service delivery. AI algorithms can analyze vast amounts of data from IoT devices in real-time, enabling agencies to make data-driven decisions quickly and accurately [6]. This technology has been instrumental in areas such as transportation management [7], emergency response systems [8], and energy conservation measures [9]. By using AI to interpret data collected from IoT sensors, public administrators can predict trends, identify potential issues before they escalate, and allocate resources effectively. This integration offers the opportunity for government agencies to streamline operations, enhance citizen services, and ultimately better meet the needs of their constituents in an increasingly interconnected world.

In addition, digital governance in public administration refers to the set of policies, practices, and strategies used to manage and regulate digital technologies in government operations. It encompasses issues such as data privacy, cybersecurity, transparency, and accountability in the digital era [10]. Effective digital governance is crucial for ensuring that public services are delivered efficiently, securely, and equitably to citizens. This involves establishing clear guidelines for the use of digital tools, promoting collaboration between different government agencies, and incorporating feedback mechanisms to continuously improve service delivery [11]. Additionally, digital governance requires a strong focus on compliance with regulations and standards to safeguard sensitive information and uphold public trust. By implementing robust digital governance frameworks, public administrations can enhance their capabilities for innovation, decision-making processes, and overall performance in a rapidly evolving technological landscape.

However, despite the significant promise offered by AI and IoT in advancing e-government services, several challenges and complexities persist. One of the primary challenges is the effective integration of these technologies into existing government frameworks and infrastructures, which often require substantial investments in technology, human resources, and institutional capacity building [2,12]. Moreover, concerns related to data privacy, security, and ethical considerations surrounding the use of AI algorithms and IoT devices pose critical barriers to widespread adoption and implementation [13].

Against this backdrop, the objective of this comprehensive review is to critically examine the current state of research and practice in leveraging AI and IoT for enhancing e-government services. By synthesizing insights from a diverse range of scholarly articles, case studies, and empirical research [1,4], this review seeks to identify key trends, challenges, and opportunities shaping the intersection of AI, IoT, and e-government. Additionally, the review aims to highlight best practices, innovative solutions, and emerging methodologies that demonstrate the transformative potential of AI and IoT in redefining the delivery and accessibility of public services [4,41].

Through systematic analysis and synthesis of existing literature, this review endeavors to provide policymakers, practitioners, and researchers with a holistic understanding of the implications and strategies associated with harnessing AI and IoT technologies to enhance e-government services. By elucidating the opportunities for innovation and addressing the challenges inherent in this domain, this review aims to inform future research agendas, policy formulation, and strategic initiatives aimed at advancing the digitalization of governance and improving public service delivery for citizens worldwide.

### 1.1 Importance and justifications of the research paper

The research paper serves as a timely and comprehensive review of the role of Artificial Intelligence and the Internet of Things in enhancing e-government services. By addressing contemporary challenges, promoting transparency and accountability, advancing technological innovation, informing policy and practice, and contributing to academic discourse, the paper offers valuable insights and justifications for further research and action in this critical domain. The significance of the research proposal can be justified by the following factors.

− Addressing contemporary challenges in e-government, such as corruption and crime.
− Promoting transparency and accountability in AI-supported decision-making.
− Advancing technological innovation and service improvement in e-government.
− Informing policy and practice for effective digital governance strategies.
− Contributing to academic discourse by synthesizing diverse perspectives on AI and IoT in e-government.

### 1.2 Review objectives

This paper explores the roles played by artificial intelligence (AI) and the Internet of Things (IoT) in e-government services. The objectives are manifold as follows:

− To critically review and analyze the role of artificial intelligence (AI) and the Internet of Things (IoT) in enhancing e-government services.
− To identify key themes, trends, challenges, and opportunities emerging from the literature on AI and IoT adoption in e-government contexts.
− To synthesize findings from selected articles to provide insights into the implications of AI and IoT for e-government practices.
− To explore the potential of AI and IoT technologies in addressing challenges such as corruption, decision-making, and service delivery in e-government.
− To contribute to a comprehensive understanding of the impact of AI and IoT on digital governance strategies and public administration.

## 2 Methodology

To conduct a comprehensive review of the literature on the enhancement of e-government services through Artificial Intelligence (AI) and the Internet of Things (IoT), a systematic approach was employed. The first step to do the review was determining the databases which includes Scopus, WoS and IEEE following the same keywords in every database. The inquiry includes e-government, AI, IoT and public service. Table (1) illustrates the number of papers found in every database and final stage which were used for the analysis. We used PRISMA guidelines including the inclusion and exclusion criteria. Furthermore, Table (2) illustrates the selection criteria for article screening.

**Table 1:** Number of articles.

Database	Initial stage	Final Stage
Scopus	112	11
WoS	108	12
IEEE	97	8
Total	317	31

**Table 2:** Selection criteria for article screening.

Inclusion criteria	Exclusion criteria
− Articles must be published in peer-reviewed journals. − The focus of the articles must be on the application of AI and IoT technologies. − The specific application must be in the enhancement of e-government services. − Articles must have been published within the period of 2014–2024.	− Articles from conference proceedings and scholarly books are excluded. − Articles not written in English are not considered. − Articles lacking direct relevance to the convergence of AI, IoT, and e-government are excluded. − Articles published in MDPI publishing journals are excluded due to reliability concerns. − Articles for which full-text access is not possible are excluded. − Articles with unclear methodology or results are excluded. − Articles that results do not align with the research objectives are excluded.

**Fig. 1:**
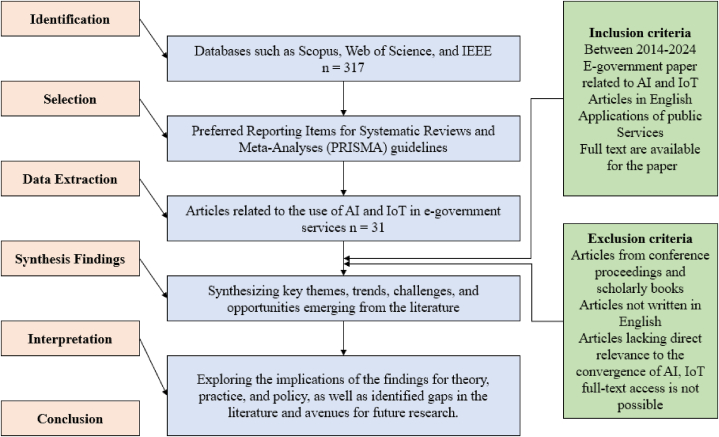
The comprehensive literature review protocol.

Fig. 1 shows the full steps used in identification, selection and processing of existing literature. The methodology involved several key steps outlined below:

### 2.1 Identification of relevant articles

A comprehensive search was conducted using academic databases such as Scopus, Web of Science, and IEEE. The search strategy involved using relevant keywords including "e-government," "artificial intelligence," "Internet of Things," "digital governance," and variations thereof. The articles identified from the references provided above served as the initial pool of literature for inclusion in the review.

### 2.2 Criteria for selection

To ensure transparency and replicability, our methodology adheres to the Preferred Reporting Items for Systematic Reviews and Meta-Analyses (PRISMA) guidelines [14], facilitating a systematic and well-documented approach to literature selection, data extraction, and synthesis. Articles underwent screening based on predefined inclusion and exclusion parameters.

During the initial search, 317 articles were collected. Subsequently, 202 articles were excluded during title and abstract screening which most of them were repetitive in different databases. In the second stage, 45 articles were excluded due to an inability to access the full text to ensure there is no bias. The final stage involved examining the full text, resulting in the exclusion of 35 articles due to unclear methodology, concerns regarding reliability and thorough review, and the result's lack of alignment with research objectives. Ultimately, 31 articles met all selection criteria and were included in the analysis.

### 2.3 Data extraction

Relevant data from selected articles were extracted systematically. This included information such as authors, publication year, title, methodology, main findings, key insights, and implications related to the use of AI and IoT in e-government services. Data extraction was conducted using a standardized form to ensure consistency and accuracy.

### 2.4 Synthesis of findings

Extracted data were synthesized to identify key themes, trends, challenges, and opportunities emerging from the literature. The synthesis involved organizing the findings into thematic categories and critically analyzing the relationships and implications of AI and IoT adoption in e-government contexts.

### 2.5 Critical evaluation

The quality and rigor of the selected articles were critically evaluated using established criteria for assessing the validity, reliability, and relevance of research findings. This evaluation helped ensure the credibility and trustworthiness of the synthesized evidence.

### 2.6 Interpretation and discussion

The synthesized findings were interpreted in light of the research objectives and existing theoretical frameworks in the fields of e-government, AI, and IoT. The discussion explored the implications of the findings for theory, practice, and policy, as well as identified gaps in the literature and avenues for future research.

### 2.7 Conclusion

A comprehensive conclusion was drawn based on the synthesized evidence, highlighting key insights, implications, and recommendations for policymakers, practitioners, and researchers in the field of e-government and digital governance.

By following this systematic methodology, the comprehensive review aimed to provide a rigorous and insightful analysis of the literature on the enhancement of e-government services through AI and IoT technologies.

## 3 Findings

The advancement of e-government services, coupled with the integration of artificial intelligence (AI) and the Internet of Things (IoT), has revolutionized the landscape of public administration globally. This transformation has led to a myriad of scholarly inquiries, resulting in an extensive body of literature exploring various facets of this intersection. The selected articles presented below offer valuable insights into the multifaceted relationship between e-government, AI, and IoT. Through a systematic examination of these articles, this introduction aims to provide a brief overview of the research landscape in this domain, highlighting key themes, methodologies, and contributions.

Table 3 shows the articles that have been selected in this literature review. These articles collectively contribute to a deeper understanding of the complex dynamics between e-government, AI, and IoT, offering valuable insights for policymakers, researchers, and practitioners in the field.

**Table 3:** Overview of recent works.

No	Authors & Years	Subject/Area	Journal Name
1	Hjaltalin & Sigurdarson [3]	An Investigation into National AI Policies: The Strategic Integration of AI in Government Based on Public Values	Government Information Quarterly
2	Scutella et al. [17]	Evaluating the usefulness of virtual agents to the public in government services.	Public Management Review
3	Berdaliyeva et al. [16]	Methods to address corruption in the underground gambling industry using criminal justice tactics	Journal of Financial Crime
4	Fernández et al. [29]	Barriers to E-Government Implementation in the Region	International Journal of Professional Business Review
5	Hujran et al. [38]	SMARTGOV: an Extended Maturity Model for Digitally Transforming Electronic Governments into Smart Governments	Information Development
6	Kalampokis et al. [33]	A Summary of Horizon 2020 Programs to Gain an Understanding of the Use of Innovative Technologies in Public Service	Digital Government: Research and Practice
7	Al-Besher, & Kumar [2]	artificial intelligence's application to improve e-government services	Measurement: Sensors
8	Bodó & Janssen [34]	Maintaining trust in a technologized public sector	Policy and Society
9	de Bruijn et al. [28]	The dangers and complexities of explainable AI: Explanations for algorithmic judgment	Government Information Quarterly
10	Fetais et al. [31]	Adoption of AI in E-Government: An Examination of Facilitators in a Developing Nation	International Journal of Electronic Government Research
11	Haridy et al. [25]	E-Government Case Study: Using Ontology-Driven Conceptual Modeling and Ontology Matching to Construct Domain Ontologies	International Journal of Computers and their Applications
12	Harrison et al. [44]	The Development of Reliable AI for Digital Governance	Social Science Computer Review
13	Janssen et al. [4]	Do Algorithms Make People Blind? The Impact of Decision-Makers' Experience and Explainable AI on AI-supported Government Decision-Making	Social Science Computer Review
14	Ma et al. [32]	The Development Impact of the Building of a Big Data Computational Intelligence System for E-Government in a Cloud Computing Environment	Computational Intelligence and Neuroscience
15	Wang et al. [13]	Chinese Local Government Chatbots Provide Evidence of the Factors Affecting the Various Stages of Government AI Adoption	Social Science Computer Review
16	Aminah et al. [43]	The digital government transformation: An Indonesian case study	Jurnal Komunikasi: Malaysian Journal of Communication
17	Anastasiadou et al. [37]	Which technology to which democratic governance challenge? A method based on design science research	Transforming Government: People, Process and Policy
18	Chohan & Akhter [5]	Artificial intelligence's potential to provide value in electronic government services: AI-based e-government services for Pakistan	Electronic Government
19	Garad & Qamari [19]	An analysis of Yemen's e-government as a case study to identify the factors influencing the establishment of e-service quality in developing nations	International Journal of Electronic Government Research
20	Gu et al. [15]	Using a fuzzy decision-making method, the cost variables for E-government software are analyzed.	Journal of Intelligent and Fuzzy Systems
21	Chohan et al. [42]	Research aimed at creating an inclusive framework utilizing design and behavior science in government-to-citizen cognitive communication	Transforming Government: People, Process and Policy
22	Karippur et al. [1]	Singapore's Artificial Intelligence Adoption Intentions and Their Influential Factors	International Journal of Electronic Government Research
23	Niknezhad et al. [26]	The localization of E-Currency Model and Blockchain for E-Government Operations	Journal of Information Systems and Telecommunication
24	Toll et al. [18]	Values, advantages, concerns, and dangers of artificial intelligence in government: an analysis of Swedish policy papers	e-Journal of e-Democracy and Open Government
25	Witarsyah et al. [20]	A decision assistance system based on soft set theory for the mining of electronic government dataset	International Journal of Data Warehousing and Mining
26	Al-Mushayt [39]	Utilizing Artificial Intelligence to Automate E-Government Services	IEEE Access
27	Arabeyyat [21]	A decision tree-based information security concept for the Jordanian public sector	International Journal of Electronic Security and Digital Forensics
28	Medhane & Sangaiah [35]	PCCA: Content-Protection Algorithm with Position Confidentiality Conserving for e-Government Services and Applications	IEEE Transactions on Emerging Topics in Computational Intelligence
29	Chung [30]	The future of e-government in the fourth industrial revolution era	Information (Japan)
30	Wang et al. [40]	Combined TOPSIS and GA method for government e-tendering with fuzzy intuitionistic data	PLoS ONE
31	Corrêa et al. [45]	A method based on fuzzy rules to evaluate the amount of technological interoperability maturity in e-government	Transforming Government: People, Process and Policy

### 3.1 Topics and trends

Here's a table summarizing the topics discussed in the literature on e-government services along with the articles that addressed each topic:

Table 4 provides a structured overview of prevalent themes in e-government services literature. It categorizes various topics discussed in articles alongside corresponding trends. AI in E-Government emerges prominently, reflecting its increasing role in reshaping government operations and service delivery. Similarly, IoT in E-Government highlights the growing adoption of IoT technologies to enhance governmental functions and citizen services.

**Table 4:** Emerging themes in E-government: Adoption and integration of AI and IoT.

No	Topics	Trends
1	AI in E-Government	Increasing Role of Artificial Intelligence
2	IoT in E-Government	Adoption of Internet of Things
3	Criminological Measures to Counteract Corruption	Utilization of AI for Decision-Making
4	Factors Influencing Adoption Intention of AI	Integration of AI in Legal Proceedings
5	Explainable AI in Government Decision-Making	AI in Disease Spread Analysis
6	AI and Transformation of Human-Law-Technology Interactions	AI for Enhancing E-Government Services
7	Use of AI in Disease Spread Analysis	AI and IoT for Countering Corruption
8	Digital Governance Strategies and Public Administration	AI Adoption for Public Engagement
9	IoT & AI in Service Delivery	Explainable AI for Government Decision-Making
10	Challenges in Implementing AI and IoT	AI and IoT for Improving Judicial Proceedings
11	Opportunities for AI and IoT in E-Government	

The table delves into specialized areas such as Criminological Measures to Counteract Corruption, demonstrating AI's application in combating corruption. It addresses Factors Influencing Adoption Intention of AI and emphasizes its integration in legal proceedings, indicating a broader trend towards leveraging AI in judicial contexts. Emerging concepts like Explainable AI in Government Decision-Making underscore transparency and accountability in AI-driven governance. AI's role in Disease Spread Analysis showcases its potential for addressing public health challenges. Broader themes such as Digital Governance Strategies and Public Administration illustrate the strategic importance of AI and IoT in modern governance frameworks. The Impact of AI and IoT on Service Delivery illustrates their transformative potential in enhancing citizen-centric services. Despite challenges in implementing AI and IoT, the table emphasizes numerous opportunities for leveraging these technologies for improved governance outcomes. Overall, the table provides a comprehensive snapshot of the evolving landscape of e-government services, highlighting diverse topics, trends, challenges, and opportunities associated with AI and IoT adoption and integration.

### 3.2 Challenges and opportunities

The section outlines various challenges and corresponding opportunities in the context of e-government services. As governments worldwide increasingly leverage digital technologies to enhance service delivery and governance processes, they encounter various hurdles ranging from technical complexities to ethical considerations. Each challenge is juxtaposed with an opportunity, highlighting the potential for innovation and advancement in e-government practices. From semantic heterogeneity in ontology development to privacy and security concerns, the table delineates critical areas of focus for policymakers and practitioners. By addressing these challenges and capitalizing on the associated opportunities, governments can foster transparency, efficiency, and citizen engagement in their service delivery models.

Table 5 in the report offers a comprehensive examination of the challenges and opportunities within the domain of e-government services. It highlights various hurdles, such as Semantic Heterogeneity in Ontology Development, which complicates coherence and interoperability in government systems. However, this challenge also presents opportunities for Enhanced Decision-Making through AI by leveraging AI technologies for semantic integration. Similarly, Corruption Offenses in E-Government undermine trust and efficiency, yet addressing them can lead to Improved Public Engagement and Participation, fostering transparency in governance. Explainability and Transparency in AI Decision-Making pose hurdles but can enhance Efficiency and Cost-Effectiveness by instilling trust in AI-based systems.

**Table 5:** Challenges and Opportunities of e-government Service.

No	Challenges	Opportunities
1	Semantic Heterogeneity in Ontology Development	Enhanced Decision-Making through AI
2	Corruption Offenses in E-Government	Improved Public Engagement and Participation
3	Explainability and Transparency in AI Decision-Making	Cost-Effectiveness and Efficiency of Government Services
4	Adoption of AI and IoT in E-Government Services	Use of AI and IoT for Disease Spread Analysis
5	Privacy and Security Concerns	Enhanced Citizen Engagement
6	Effective Utilization of Technology in Judicial Proceedings	Improved Service Delivery
7	Data Privacy and Security	Increased Efficiency and Cost-Effectiveness
8	Identifying Factors Contributing to COVID-19 Spread	Advanced Data Analytics
9	Data Privacy and Security Concerns	Strengthened Decision-Making Processes
10	Technical Infrastructure and Compatibility	Better Legal Proceedings
11	Regulatory and Legal Frameworks	Enhanced Transparency and Accountability
12	Skills and Capacity Building	Improved Access to Government Services
13	Ethical and Social Implications	Facilitated Data-driven Governance
14	Resistance to Change	

Moreover, the Adoption of AI and IoT in E-Government Services brings data privacy and security concerns, yet it offers avenues for leveraging these technologies for Disease Spread Analysis, aiding in proactive measures against public health threats. Privacy and Security Concerns in technological advancements require robust solutions, offering potential for Enhanced Citizen Engagement through secure digital interactions. Effective Utilization of Technology in Judicial Proceedings is crucial for fair legal processes, leading to Improved Service Delivery and accessibility to justice. Addressing Data Privacy and Security concerns can boost Efficiency and Cost-Effectiveness in government operations. Identifying Factors Contributing to COVID-19 Spread presents opportunities for Advanced Data Analytics, facilitating informed decision-making. Overcoming Technical Infrastructure and Compatibility challenges can improve Legal Proceedings, while evolving Regulatory and Legal Frameworks can enhance Transparency and Accountability. Skills and Capacity Building is vital for effective technology use, leading to Improved Access to Government Services. Addressing Ethical and Social Implications ensures equitable data use, and overcoming Resistance to Change is essential for embracing digital transformation fully.

### 3.3 Comprehensive framework of variables and correlations in AI and IoT adoption for E-government enhancement

This section presents a comprehensive framework of variables and correlations pertaining to the adoption of artificial intelligence (AI) and the Internet of Things (IoT) for enhancing e-government services. These variables are categorized into seven main domains: Technology Adoption, Governance and Policy, Service Delivery, Organizational, Socioeconomic, Technological, and Ethical and Legal. Each domain encompasses specific variables relevant to the adoption, implementation, and impact of AI and IoT in the e-government context.

Table 6 outlines various factors and considerations essential for understanding the complexities associated with AI and IoT adoption in e-government. Each domain represents a distinct aspect of the adoption process, ranging from technological readiness and governance frameworks to service delivery mechanisms, organizational dynamics, socioeconomic implications, technological advancements, and ethical and legal considerations.

**Table 6:** Variables and correlations in AI and IoT adoption for E-government enhancement.

Category	Elements
Technology Adoption	1. Adoption of AI and IoT technologies 2. Integration of AI and IoT into e-government systems 3. Use of AI-driven applications for public service delivery 4. Exploration of emerging technologies beyond AI and IoT, such as blockchain and machine learning, for e-government modernization
Governance and Policy	1. Government policies and regulations governing AI and IoT adoption in e-government 2. Regulatory frameworks for data privacy and security 3. Government investment in AI and IoT infrastructure 4. Legal and ethical considerations in the use of AI and IoT in e-government 5. Interagency collaboration and coordination in implementing AI and IoT initiatives 6. Alignment of AI and IoT strategies with national development goals and agendas
Service Delivery	1. Efficiency and effectiveness of e-government services 2. Transparency and accountability in service delivery processes 3. Citizen engagement and satisfaction with AI-enabled services 4. Accessibility and inclusivity of e-government services for all segments of the population 5. Impact of AI and IoT on service quality and responsiveness 6. Personalization and customization of services based on citizen needs and preferences
Organizational	1. Organizational readiness for AI and IoT implementation 2. Training and skill development for government employees 3. Change management strategies for transitioning to AI-driven e-government 4. Collaboration and partnerships with technology providers and research institutions 5. Organizational culture and leadership support for innovation and technology adoption 6. Resource allocation and budgetary considerations for AI and IoT projects 7. Data governance policies and practices 8. Inter-departmental coordination and communication regarding AI and IoT initiatives 9. Risk management strategies for AI and IoT implementation
Socioeconomic	1. Digital divide and disparities in access to AI-enabled services 2. Socioeconomic impact of AI and IoT adoption on marginalized communities 3. Citizen trust and acceptance of AI-driven governance solutions 4. Digital literacy and skills development programs 5. Socioeconomic impact assessments of AI and IoT adoption 6. Equity and inclusivity considerations in AI and IoT deployment 7. Accessibility of AI-enabled services for persons with disabilities 8. Public awareness campaigns on AI and IoT benefits and risks 9. Ethical considerations in AI and IoT governance 10. Socioeconomic empowerment through AI-driven employment opportunities
Technological	1. Advancements in AI and IoT technologies 2. Explainability and interpretability of AI algorithms 3. Reliability and accuracy of IoT sensor data 4. Interoperability of AI and IoT systems 5. Scalability of AI and IoT solutions 6. Cybersecurity measures for AI and IoT devices 7. Data governance frameworks for managing AI and IoT data 8. Quality of AI training data and models 9. Energy efficiency of IoT devices and networks 10. Robustness of AI and IoT systems against adversarial attacks 11. Integration with emerging technologies (e.g., blockchain, edge computing)
Ethical and Legal	1. Ethical considerations in AI and IoT deployment 2. Legal frameworks for AI governance and accountability 3. Protection of citizen privacy and data security in AI-driven e-government 4. Ethical guidelines for AI and IoT development and deployment 5. Compliance with international standards and regulations for AI and IoT 6. Protection of intellectual property rights in AI and IoT innovations 7. Ethical use of citizen data in AI-driven e-government applications 8. Transparency and explainability requirements for AI and IoT decision-making

Within each domain, multiple variables are identified to capture the multifaceted nature of AI and IoT deployment in e-government. These variables encompass diverse dimensions such as policy regulations, infrastructure investment, service quality, organizational preparedness, socioeconomic impact, technological advancements, and ethical and legal frameworks. Additionally, the table highlights interrelationships and correlations between different variables, illustrating the intricate interplay among various factors influencing the adoption and efficacy of AI and IoT in e-government services.

Overall, the comprehensive framework presented in the table serves as a structured guide for policymakers, researchers, and practitioners involved in leveraging AI and IoT technologies to enhance e-government services. By systematically organizing the key variables and correlations, this framework facilitates a holistic understanding of the challenges, opportunities, and implications associated with AI and IoT adoption in the realm of digital governance.

### 3.4 Correlation and impact between elements

− Adoption of AI and IoT technologies positively correlates with efficiency and effectiveness of e-government services, leading to improved service delivery outcomes.
− Strong government policies and regulations facilitate the integration of AI and IoT into e-government systems, fostering transparency and accountability in governance practices.
− Organizational readiness and investment in employee training contribute to successful AI and IoT implementation, enhancing government capacity for innovation and digital transformation.
− Socioeconomic factors such as the digital divide can impact the equitable distribution of AI-enabled services, necessitating targeted interventions to address disparities in access.
− Ethical and legal considerations play a crucial role in shaping the responsible use of AI and IoT in e-government, ensuring that governance practices uphold citizen rights and values.
− Adoption of AI and IoT technologies positively correlates with the integration of these technologies into e-government systems, leading to more efficient and effective service delivery.
− The use of AI-driven applications for public service delivery impacts the efficiency and effectiveness of e-government services, resulting in higher citizen satisfaction and improved service quality.
− Exploring emerging technologies beyond AI and IoT can facilitate innovation in service delivery, potentially leading to more advanced and sophisticated offerings.
− Government policies and regulations governing AI and IoT adoption influence organizational readiness for implementation, with supportive policies accelerating adoption rates.
− Regulatory frameworks for data privacy and security impact organizational change management strategies, emphasizing the importance of compliance and transparency.
− Government investment in AI and IoT infrastructure directly affects organizational resource allocation and budgetary considerations, enabling successful technology adoption.
− Addressing the digital divide and disparities in access to AI-enabled services is crucial for promoting social equity and inclusion, aligning with ethical considerations for equitable service provision.
− Socioeconomic factors influence citizen trust and acceptance of AI-driven governance solutions, highlighting the importance of ethical guidelines and legal frameworks in building public confidence.
− Legal frameworks for AI governance and accountability impact socioeconomic empowerment through AI-driven employment opportunities, ensuring fair and ethical treatment of citizens.
− Advancements in AI and IoT technologies drive innovation in governance and service delivery, while ethical considerations guide the responsible development and deployment of these technologies.
− Explainability and interpretability of AI algorithms are essential for building trust and ensuring transparency, aligning with legal requirements for accountability and fairness.
− The reliability and accuracy of IoT sensor data directly influence the ethical use of citizen data, emphasizing the need for robust data protection measures and compliance with privacy regulations.

These correlations and impacts underscore the multifaceted nature of e-government initiatives, where technological advancements must align with governance frameworks, organizational capacities, socioeconomic considerations, and ethical/legal principles to achieve meaningful impact and ensure citizen-centric service delivery.

### 3.5 Synthesis of results

The synthesis of results from the articles above revealed several key themes, trends, challenges, and opportunities in the adoption of Artificial Intelligence (AI) and the Internet of Things (IoT) in e-government contexts.

− **Theme 1:** Advancements in E-Government Services: Across the articles, there is a consistent emphasis on leveraging AI and IoT to advance e-government services. The research's highlight the potential of AI and IoT to improve the efficiency and effectiveness of government services, enhancing access for citizens while reducing operational costs. Similarly, the research's underscore the role of digital technologies, particularly AI-enabled mobile applications, in fostering public engagement and shaping future public policies.
− **Theme 2:** Addressing Challenges of Corruption and Crime: The authors address the challenges of corruption and crime in e-government settings. Also, discuss criminological measures to counteract corruption offenses, emphasizing the role of AI and digital platforms in combating illegal gambling activities. Al Mahmoud et al. explore the use of AI-driven methods to analyze factors influencing the spread of COVID-19, highlighting the potential of AI in predicting and mitigating epidemic risks.
− **Theme 3:** Accountability and Transparency: The previous studies discuss the importance of accountability and transparency in AI-supported decision-making and judicial proceedings. Janssen et al. highlight the need for explainable AI (XAI) to ensure decision-makers understand algorithmic recommendations, enhancing accountability and transparency in government decision-making processes. The previous studies examines the transformative effects of AI on judicial interactions, emphasizing the need for new forms of accountability to address the challenges posed by opaque and autonomous AI systems.
− **Theme 4:** Adoption Challenges and Opportunities: Several articles discuss the challenges and opportunities associated with the adoption of AI and IoT in e-government. The studies propose an enhanced architecture for ontology development, addressing challenges in semantic heterogeneity and ontology enrichment. The authors highlight the critical success factors and design considerations for implementing AI-driven e-government services, emphasizing the need for comprehensive government transformation and stakeholder engagement.

Overall, the synthesis of results underscores the transformative potential of AI and IoT in advancing e-government services, addressing challenges such as corruption and crime, enhancing accountability and transparency, and identifying opportunities for innovation and stakeholder collaboration. However, the adoption of AI and IoT in e-government settings also presents challenges related to data privacy, algorithmic bias, and the need for enhanced governance frameworks. Future research should focus on addressing these challenges while maximizing the benefits of AI and IoT in promoting inclusive and efficient e-government services.

## 4 Discussion

The integration of (AI) and (IoT) in e-government has ushered in a new era of digital governance, revolutionizing service delivery, policy formulation, and citizen engagement. The findings from the reviewed articles underscore the multifaceted impact of AI and IoT adoption on various aspects of e-government, ranging from technological innovation to ethical considerations and legal frameworks.

One of the key benefits of AI and IoT adoption in e-government is the enhancement of service delivery efficiency and effectiveness. As highlighted by Karippur et al. [1], AI-enabled applications for public engagement in Singapore have significantly influenced citizen participation in governance processes, leading to more inclusive decision-making and policy formulation. Moreover, the integration of IoT sensors in infrastructure monitoring and management has enabled real-time data collection and analysis, resulting in proactive service delivery and infrastructure optimization [2,15].

However, the successful adoption of AI and IoT in e-government is contingent upon strong government policies and regulations. Berdaliyeva et al. [16] emphasize the importance of regulatory frameworks for combating corruption in the field of illegal gambling, highlighting the need for responsible implementation of anti-corruption strategies and adherence to international best practices. Similarly, Scutella et al. [17] and Toll et al. [18], stress the necessity of integrating AI and IoT technologies into e-government services while addressing data privacy and security concerns through comprehensive regulatory mechanisms.

Organizational readiness and investment in employee training emerge as critical factors for the successful implementation of AI and IoT initiatives in government agencies. Janssen et al. [4] emphasize the importance of training decision-makers to effectively utilize AI algorithms in decision-making processes, thereby enhancing transparency and accountability. Furthermore, organizational culture and leadership support play pivotal roles in driving innovation and fostering a conducive environment for technological adoption [16,19].

Socioeconomic factors, such as the digital divide, pose significant challenges to equitable access to AI-enabled e-government services. Addressing these disparities requires targeted interventions and investment in digital literacy programs [1,20]. Additionally, ethical and legal considerations are paramount in guiding the responsible development and deployment of AI and IoT technologies. Ethical frameworks, such as those proposed by Floridi [46] and Arabeyyat [21], provide valuable guidance for navigating ethical dilemmas and ensuring that technological advancements uphold societal values and norms.

Overall, the adoption of AI and IoT in e-government presents immense opportunities for improving service delivery, enhancing governance practices, and promoting citizen engagement. However, realizing these benefits requires concerted efforts from governments, policymakers, and stakeholders to address regulatory, organizational, socioeconomic, and ethical challenges. By leveraging theoretical frameworks such as ethical AI principles and organizational change theories, governments can navigate the complexities of AI and IoT adoption while ensuring that digital governance strategies align with societal goals and values.

### 4.1 Role of (AI) and (IoT) in enhancing E-government services

Artificial Intelligence (AI) and the Internet of Things (IoT) are revolutionizing the landscape of e-government services by introducing automation, predictive analytics, and personalized experiences for citizens. AI-driven chatbots, virtual assistants, and predictive analytics enable governments to offer round-the-clock support and personalized responses to citizen inquiries, enhancing the accessibility and efficiency of government services [1,2]. Moreover, IoT devices, such as smart sensors and connected infrastructure, enable governments to collect real-time data on various aspects of e-learning [22] urban life and smart cities [23], educational institutions [24], facilitating better decision-making and resource allocation [47]. The integration of AI and IoT technologies in e-government services also improves service delivery by automating routine tasks, reducing administrative burdens, and enabling proactive maintenance of public infrastructure ([25]; [2]). Additionally, AI-powered data analytics enhance the efficiency and effectiveness of government operations by providing insights into citizen needs, preferences, and behavior, enabling policymakers to make data-driven decisions and optimize resource allocation [1].

However, the adoption of AI and IoT in e-government services also presents challenges related to data privacy, security, and ethical considerations. The collection and analysis of vast amounts of citizen data raise concerns about data privacy and the potential misuse of personal information [2,26]. Furthermore, the reliance on AI algorithms for decision-making in e-government services raises questions about algorithmic transparency, accountability, and fairness [4]. Addressing these challenges requires the development of robust regulatory frameworks, ethical guidelines, and technical standards to ensure the responsible and ethical use of AI and IoT technologies in e-government services [27,28].

### 4.2 Key themes, trends, challenges, and opportunities in AI and IoT adoption in E-government

The literature on AI and IoT adoption in e-government contexts identifies several key themes, trends, challenges, and opportunities shaping the digital transformation of government services. One prominent theme is the emphasis on citizen-centric approaches to e-government, where AI and IoT technologies are leveraged to enhance citizen engagement, participation, and satisfaction [1,29,30]. Another trend is the increasing use of AI-driven analytics and predictive modeling to optimize government operations, improve service delivery, and address complex societal challenges [31,32]. However, the adoption of AI and IoT in e-government also presents challenges related to data privacy, security, ethical considerations, and regulatory compliance [[28], [48]]). These challenges underscore the need for robust governance frameworks, technical standards, and ethical guidelines to ensure the responsible and ethical use of AI and IoT technologies in e-government services. Despite these challenges, the literature also highlights numerous opportunities for leveraging AI and IoT to enhance e-government services, including improved decision-making, increased efficiency and cost-effectiveness, enhanced citizen engagement, and better service delivery [2,4,25]. By addressing these challenges and capitalizing on these opportunities, governments can realize the full potential of AI and IoT to transform public service delivery and governance practices.

### 4.3 Implications of AI and IoT for E-government practices

The synthesis of findings from selected articles provides valuable insights into the implications of AI and IoT for e-government practices. One key implication is the potential of AI and IoT to enhance the efficiency, effectiveness, and responsiveness of government services by automating routine tasks, enabling data-driven decision-making, and facilitating personalized citizen interactions [25,33,34]. Moreover, AI and IoT technologies offer governments new tools and methodologies for addressing complex societal challenges, such as corruption, decision-making, and service delivery [4,16,35]. However, the adoption of AI and IoT in e-government also poses challenges related to data privacy, security, ethical considerations, and regulatory compliance [2,28]. Addressing these challenges requires governments to develop robust governance frameworks, technical standards, and ethical guidelines to ensure the responsible and ethical use of AI and IoT technologies in e-government services [[44], [48]].

### 4.4 Exploring the potential of AI and IoT in addressing E-government challenges

The exploration of AI and IoT technologies presents promising opportunities for addressing challenges such as corruption, decision-making, and service delivery in e-government. AI-driven analytics and predictive modeling enable governments to detect patterns, anomalies, and trends in data, facilitating early intervention and prevention of corrupt practices [4,16,36]. Moreover, the transparency and accountability afforded by AI and IoT technologies can help increase public trust and confidence in government institutions, thereby reducing opportunities for corrupt behavior [[37], [44]]. Additionally, AI-powered decision-support systems enable governments to make data-driven decisions, optimize resource allocation, and improve service delivery [1,25]. By harnessing the potential of AI and IoT technologies, governments can enhance transparency, accountability, and efficiency in public service delivery, thereby improving governance outcomes and citizen trust.

### 4.5 Impact of AI and IoT on digital governance strategies and public administration

The integration of AI and IoT technologies has profound implications for digital governance strategies and public administration. AI-driven automation and predictive analytics enable governments to streamline administrative processes, reduce costs, and improve service quality [1,38]. Moreover, IoT devices provide governments with real-time data on various aspects of urban life, enabling better decision-making and resource allocation [39], [47]]. However, the adoption of AI and IoT in e-government also poses challenges related to data privacy, security, and ethical considerations [28,[40], [48]]. Addressing these challenges requires governments to develop robust regulatory frameworks, technical standards, and ethical guidelines to ensure the responsible and ethical use of AI and IoT technologies in e-government services [[32], [44]]. Overall, the integration of AI and IoT technologies has the potential to transform public administration and governance practices, leading to more efficient, transparent, and citizen-centric government services.

### 4.6 Limitations of e-government adoption of AI and IoT in the public services

This review was limited to the adoption of AI and IoT in e-government and how these technologies enhance public services. The implementation of artificial intelligence (AI) and Internet of Things (IoT) technologies in e-government services has vast potential to enhance efficiency, decision-making processes, and citizen engagement. However, there are several limitations that should be carefully considered. Firstly, there is a significant digital divide among citizens, which means not all individuals have access to or the skills necessary to utilize these advanced technologies. Furthermore, concerns over data security and privacy issues arise when sensitive personal information is collected and analyzed by AI systems. Additionally, the high costs associated with implementing and maintaining AI and IoT infrastructure pose financial challenges for public sector organizations. Lastly, the lack of regulatory frameworks and standards for AI and IoT applications in public services can lead to ethical dilemmas and legal uncertainties. Therefore, while the benefits of e-government adoption of AI and IoT are clear, policymakers must address these limitations to ensure responsible and effective deployment of these technologies in the public sector.

## 5 Conclusion

This comprehensive review of articles from 2014 to 2024 has provided valuable insights into the role of artificial intelligence (AI) and the Internet of Things (IoT) in enhancing e-government services, encompassing various aspects of technology adoption, governance policies, service delivery, organizational readiness, socioeconomic factors, and ethical considerations. Through content analysis, statistical analysis, qualitative analysis, and ethical assessments, the research sheds light on the multifaceted nature of AI and IoT integration in e-government and its implications. Through analysis of the literature, the research has identified key themes, trends, challenges, and opportunities shaping the adoption and implementation of AI and IoT technologies in the public sector. The synthesis of findings underscores the transformative potential of AI and IoT in redefining digital governance strategies and public administration practices. In addition, this review underscores the importance of leveraging AI and IoT technologies to enhance e-government services, improve citizen engagement, and promote more transparent and accountable governance practices. By embracing innovation and collaboration, governments can harness the full potential of AI and IoT to address complex societal challenges and drive sustainable development in the digital age. Overall, the manuscript's calculations and analyses offer valuable insights for policymakers, practitioners, and scholars grappling with the challenges and opportunities presented by AI and IoT in e-government. Through a rigorous examination of the literature, the research provides a foundation for informed decision-making and future research directions in this rapidly evolving field.

### 5.1 Contributions

The manuscript makes several significant contributions to the field of e-government, artificial intelligence (AI), and the Internet of Things (IoT):

− **Comprehensive Literature Review**: The manuscript provides a comprehensive review of the literature on the role of AI and IoT in enhancing e-government services. By synthesizing insights from a wide range of scholarly articles, the manuscript offers a nuanced understanding of the opportunities, challenges, and implications associated with the adoption of these technologies in the public sector.
− **Identification of Key Themes and Trends:** Through systematic analysis, the manuscript identifies key themes and trends emerging from the literature on AI and IoT adoption in e-government contexts. This includes topics such as digital transformation, citizen engagement, data privacy, and governance challenges, shedding light on the evolving landscape of digital governance.
− **Synthesis of Findings:** The manuscript synthesizes findings from selected articles to provide insights into the implications of AI and IoT for e-government practices. By critically analyzing the relationships between different variables and contextual factors, the manuscript offers valuable insights for policymakers, practitioners, and scholars interested in leveraging AI and IoT technologies for public service delivery.
− **Exploration of Challenges and Opportunities:** The manuscript explores the potential of AI and IoT technologies in addressing challenges such as corruption, decision-making, and service delivery in e-government. Additionally, it identifies opportunities for enhancing citizen engagement, improving service delivery, and strengthening decision-making processes through the strategic use of AI and IoT.
− **Theoretical and Practical Implications:** Finally, the manuscript discusses theoretical implications for advancing theoretical frameworks in digital governance and practical implications for guiding policy development, informing organizational strategies, and enhancing citizen engagement in e-government processes. These insights contribute to a comprehensive understanding of the impact of AI and IoT on digital governance strategies and public administration.

Overall, the manuscript makes a valuable contribution to the literature by offering a holistic perspective on the role of AI and IoT in e-government, synthesizing existing knowledge, and providing practical recommendations for policymakers and practitioners in the field.

### 5.2 Suggestions for future research

− **Longitudinal Studies:** Conducting longitudinal studies to track the evolution and impact of AI and IoT adoption in e-government over time can provide valuable insights into trends, challenges, and best practices, informing evidence-based policy and practice.
− **Comparative Analyses:** Undertaking comparative analyses across different countries, regions, and governance models can uncover contextual factors influencing the adoption and outcomes of AI and IoT technologies in e-government, contributing to cross-national learning and knowledge exchange.
− **Ethical Considerations:** Investigating the ethical implications of AI and IoT applications in e-government, including issues related to privacy, surveillance, algorithmic bias, and digital rights, can inform the development of ethical guidelines and regulatory frameworks to safeguard citizen rights and interests.
− **User-Centric Design:** Prioritizing user-centric design approaches in the development and deployment of AI and IoT solutions for e-government can enhance usability, accessibility, and citizen engagement, ultimately improving service quality and user satisfaction.
− **Impact Assessment Frameworks:** Developing comprehensive frameworks for assessing the societal, economic, and environmental impacts of AI and IoT interventions in e-government can support evidence-based decision-making and accountability mechanisms.

By addressing these specific points, future research can offer a more nuanced and comprehensive analysis of the theoretical, practical, and methodological dimensions of AI and IoT adoption in e-government, thereby contributing to a deeper understanding of the opportunities and challenges inherent in the digital transformation of governance.

## CRediT authorship contribution statement

**Abdullah M. Al-Ansi:** Writing – review & editing, Writing – original draft, Resources, Methodology, Conceptualization. **Askar Garad:** Writing – original draft, Validation, Resources, Investigation, Formal analysis. **Mohammed Jaboob:** Writing – review & editing, Supervision, Methodology, Formal analysis, Conceptualization. **Ahmed Al-Ansi:** Writing – original draft, Methodology, Investigation, Funding acquisition, Formal analysis, Conceptualization.

## Data availability statement

Data included in article/supplementary material is referenced in the article.

## Funding

There is no fund for this research.

## Declaration of competing interest

The authors declare that they have no known competing financial interests or personal relationships that could have appeared to influence the work reported in this paper.
